# Atypical Systemic Leishmaniasis to Be Considered in the Differential of Patients Presenting with Depressed Immunity

**DOI:** 10.1371/journal.pntd.0001782

**Published:** 2012-08-21

**Authors:** Nuha Nuwayri-Salti, Khouzama Knio, Adham Jammoul, Rajaa Fakhoury, Karim A. Sarhane, Hania Nakkash-Chmaisse

**Affiliations:** 1 Department of Human Morphology, Faculty of Medicine, American University of Beirut, Beirut, Lebanon; 2 Department of Pediatrics, Rafic Hariri University Hospital, Beirut, Lebanon; 3 Department of Biology, Faculty of Arts and Sciences, American University of Beirut, Beirut, Lebanon; 4 Department of Neurology, Neurological Institute, Cleveland Clinic Foundation, Cleveland, Ohio, United States of America; 5 Biological and Environmental Science Department, Faculty of Science, Beirut Arab University, Beirut, Lebanon; 6 Department of Biochemistry and Molecular Genetics, Faculty of Medicine, American University of Beirut, Beirut, Lebanon; 7 Department of Pharmacology, Faculty of Pharmacy, Beirut Arab University, Beirut, Lebanon; Emory University, United States of America

## Abstract

**Background:**

Systemic leishmaniasis has been known to present with prolonged fever, hepatosplenomegaly and wasting. Beside this classical form, a sub-clinical form has been identified. It is described with either one or two of the above symptoms missing; other findings have been reported instead, such as lymphadenopathy and anemia. In this report, we reveal a third unsuspected form which we are referring to as “atypical”.

**Methodology/Principal Findings:**

Patients suspected to be immune-deficient were referred to our immunology specialized laboratory to study some aspects of their immune functions (not normally covered in the general laboratory). Multiple specialized tests were performed, including microscopic examinations using appropriate stains, and mainly cultures of biopsies on several types of specialized media. 19·4% of 160 patients were found to have close to normal laboratory profiles, but exhibited dysfunctional macrophages laden with *Leishmania* parasites.

**Conclusions/Significance:**

Findings such as the ones we obtained allowed us to uncover the presence of patients with an atypical form of systemic leishmaniasis. It presents with symptoms masquarading a condition in which the immune system is non functional. This predisposes patients to recurrent secondary infections resulting in clinical pictures with a great variety of signs and symptoms. These findings alerted us to the fact that systemic leishmaniasis presents with a much wider spectrum of signs and symptoms than so far suspected and is far more common than diagnosed to date. Furthermore, among these 31 patients was a number of adults. This proved that in our area systemic leishmaniasis is surely not limited to the pediatric age group. Our recommendation is to entertain the diagnosis of atypical systemic leishmaniasis in any patient with an unexplained depressed immunity state and in whom no obvious immunologic defect can be identified.

## Author Summary

Systemic leishmaniasis is known to have in general two types of clinical presentations. The classical form is a full fledged disease characterized by fever, severe weight loss, enlargement of lymph organs and anemia. It renders patients very sick and is sometimes fatal. The second form, known as sub-clinical leishmaniasis, is a milder disorder. Although it presents with symptoms analogous to the ones classically associated with this infection, they are far less severe and certainly fewer in number. In this report, we are describing a third type of presentation, which is distinct from the above described. The hallmark of this third type of clinical form is depressed immunity. A number of patients suffering from repeated infections affecting various body organs and systems (skin, respiratory, and alimentary) were referred to us for advanced immune function testing. We were able to identify in a fraction of these patients *Leishmania parasites*. Upon appropriate treatment, these patients recovered. Systemic leishmaniasis was thus the culprit of their repeated episodes of falling ill. Hence our recommendation in this report is to consider the diagnosis of systemic leishmaniasis in patients suffering from signs and symptoms indicating depressed immunity (repeated episodes of fever or infections).

## Introduction

Visceral Leishmaniasis (VL) or Kala-Azar was first described by Leishman in 1906. It is characterized by fever (periodic or continuous), cachexia, organomegaly (liver and spleen) causing the abdomen to protrude, severe wasting of the limbs and trunk, and pancytopenia [1]–[4]. Due to systemic parasite dissemination, the disease is in general fatal if untreated [5].

Reviewing the literature, there seems to exist two types of systemic leishmaniasis. A form presenting with the above described signs and symptoms i.e. a classical form, and another type of the disorder referred to as the sub-clinical. The latter was first alluded to by Leishman in 1906; then it was followed by several groups, starting from the late 1950s with Manson-Bahr [6], who recognized the presence of infected subjects carrying the parasite yet presenting with symptoms far milder. All of these groups based their diagnosis on positive immunological responses to the parasite [7]–[16].

The ratio of patients with sub-clinical disease to those with overt classical symptoms varied from one area to the other, and from one year to another. Its value (sub-clinical/overt) shifted from levels as low as 1∶2·4 to values as high as 18·5∶1 [14]. Part of the discrepancy in the reported figures is the result of several factors: First, the lack of agreement in defining this disorder; second, the limitations of the varied diagnostic tools, and third the geographic and demographic differences among populations (e.g. nutrition, genetics) [11], [14], [17].

In all cases, diagnosis of systemic leishmaniasis was dependent principally on the immune reaction to *Leishmania* antigens, especially that tissue examination does not always successfully demonstrate the parasite, neither do cultures of specimens from affected tissues.

As to our area, Bitar and Nachman reported 72 pediatric cases of leishmaniasis from five hospitals in Beirut (Lebanon). They were discovered between 1926 and 1964 (about 2 cases per year) [18]. Visceral Leishmaniasis presented then with symptoms and signs consistent with the typical textbook picture. This pathology invariably ended by the lymphatic system's insufficiency, hence total immune paralysis and death of the patient.

Over the last 13 years, 160 patients diagnosed with depressed immunity, and who underwent exhaustive laboratory investigations to no avail, were referred to our specialized laboratory for further evaluation of their immune system function.

Hence our objective in this report is to disclose to scientists in the field that systemic leishmaniasis has a clinical picture with far more protean signs and diverse symptoms.

## Materials and Methods

### Ethics Statement

This project received approval from the Institutional Review Board of the American University of Beirut Medical Centre (AUB–MC) as part of our epidemiologic surveillance study initiated in 1994, when the *Leishmania* study group was established. The project objectives were re-approved in 1999. We obtained written informed consent from all patients or from their parents/guardians (when they were less than 18 years of age). It was agreed not to reveal the identity of the patients should the results of their laboratory tests be used in any research study.

### Patients and Study Design

We reviewed all the cases referred to our laboratory situated at AUB–MC with the diagnosis of depressed immunity. Part of our routine in these cases was a questionnaire answered by each patient. We extracted the biodata with special attention to the major clinical features in the symptomatology. We also included the geographic area where the patients lived and live, as well as their personal and family histories. Travel in and outside the area was recorded as well.

As for the battery of laboratory tests carried out on every patient, these consisted of a complete blood count (CBC), immunoglobulin titers (total and subclasses), complement activity and titers (total, C3 and C4). In addition, we tested chemotactic activity using a Skin Window. That was performed at 2 time intervals, one slide at 4 hours for the acute response, and the other slide at 18 hours for the chronic response [19]. In suspicious cases, the window was modified to detect the presence of monocytes with *leishmania* organisms (report in preparation). Whenever the cellular response on this test was deficient and in the absence of any infectious agents affecting the cells, a Boyden chamber assay was performed to analyze the defect revealed by the Skin Window.

In addition, blood cultures on classical media (bacteria and fungi) and on Novy Nicolle McNeal medium (NNN medium) were also carried out.

The majority of patients had sections of biopsies from either a lymph node or a bone marrow, or had an aspirate of bone marrow and less often of the spleen. All of these samples were obtained in the course of the general investigation of their “defective” immunity and often their anemia. Available sections and/or aspirates were stained with leishman and acid fast stains for microscopic examination. Immunofluorescence for *Leishmania* parasites was used for confirmation in equivocal cases. For the latter, a positive control from axenic parasite cultures was always included.

## Results

### Patients' Analysis

31 of the 160 referred patients (19·4%) were found to suffer from an infection with *Leishmania* parasites. Presentation of these subjects and behavior of the parasite in this group constitute the focus of the current report.

**Figure 1:**
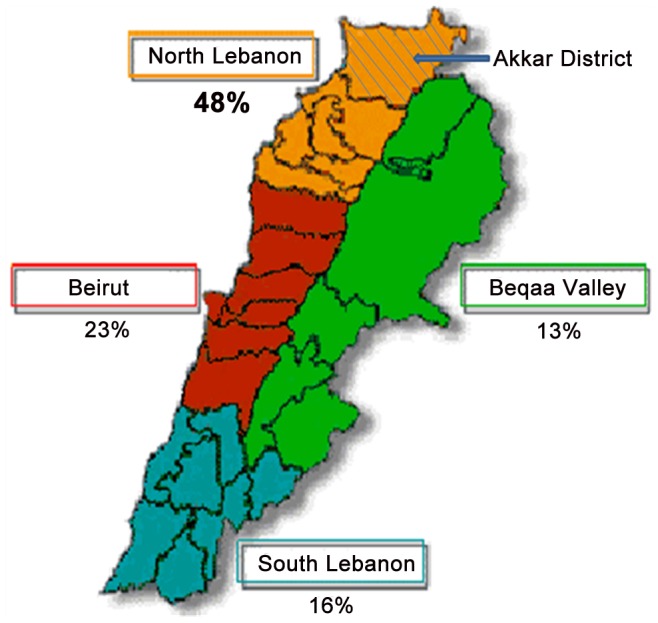
Geographical distribution of the presenting patients.

The majority of the infected patients were from the North of Lebanon (48%), Akkar District, which was identified as a focus in 1999–2000 [20]. This locale was followed by Beirut area (23%) then by the South (16%) and last the Beqaa Valley (13%) (Figure 1).

The age at presentation of these cases ranged from a few months to 45 years. The majority by far (84·8%) were children in the age group between 2 months and 12 years, with a bimodal peak around 2 and 6 years. Worth stressing that there were a few adults. The gender was equally distributed, 16 males and 15 females. Duration of the presenting illness spanned from a few months to 11 years, in most cases lasting between 2 and 3 years. The symptoms varied in severity and nature of the targeted organ.

This biodata, including the geographic locale, age at presentation, gender, duration of illness in addition to the major clinical problems that brought the patient, is summarized in table 1.

**Table 1:** Salient biodata of the 31 patients.

Patients	Age at Presentation (years)	Gender	Major Clinical Problem	Duration	Geographic Locale
1	7	F	Failure to thrive	4 years	North
2	5	M	Recurrent infections (different systems)	2 years	North
3	8	M	Recurrent diarrhea	5 years	Beirut
4	28	F	Recurrent subcutaneous boils	1 year	North
5	2	F	Recurrent diarrhea and failure to thrive	17 months	Beirut
6	8	F	Pallor with poor energy	3 years	Beirut
7	3	F	Failure to thrive	3 years	South
8	41	F	High grade fever on and off with poor energy	2 years	South
9	7	M	Low grade fever with splenomegaly and hypogammaglobulinemia	18 months	North
10	6	M	Recurrent upper respiratory tract infections	3 years	North
11	8	F	Recurrent subcutaneous boils	4 years	North
12	8	M	Failure to thrive	3 years	North
13	10	F	Low grade fever on and off, then continuous over several weeks	6 months	Beqaa
14	9	M	Recurrent subcutaneous boils	1 year	Beqaa
15	12	F	Growth arrest at age 7	5 years	North
16	7	M	Recurrent subcutaneous boils	10 months	Beirut
17	7	M	Recurrent subcutaneous boils	3 years	North
18	5	M	Recurrent infections (different systems)	3 years	Beirut
19	2	M	Recurrent infections (different systems)	23 months	Beqaa
20	45	F	Low grade fever on and off, then continuous over the last 3 months	11 months	South
21	8	M	Recurrent diarrhea	14 months	South
22	3	M	Recurrent subcutaneous boils	20 months	Beqaa
23	10	M	Recurrent subcutaneous boils	4 years	South
24	6	M	Recurrent diarrhea	3 years	North
25	9	F	Slow growth and poor energy	6 years	North
26	8	M	Recurrent subcutaneous boils	2 years	Beirut
27	36	F	Recurrent subcutaneous boils	16 months	Beirut
28	13	M	Low grade fever on and off	16 months	North
29	5	F	Recurrent subcutaneous boils	2 years	North
30	12	F	Low grade fever on and off with recurrent diarrhea	11 years	North
31	36	F	Low grade fever with splenomegaly	4 months	North

The most frequent complaint was recurrent infections (74·2%) expressed by pathology either in the skin, the gastrointestinal tract, or the respiratory system. Skin infections (subcutaneous boils and/or deep abscesses), occurring anywhere on the body, were in the lead. To note, these were devoid of any detectable insect bite site.

Failure to thrive and Fever of Unknown Origin (FUO) came second in frequency. Splenomegaly was found in the minority of cases (Table 2).

**Table 2:** Rates of occurrence of various symptoms.

Presenting symptoms	# of cases	Rate %
Frequent Infections	23/31	74·2
<$>\raster="rg1"<$> Skin (boils/deep abscesses)	12/23	52·2
<$>\raster="rg1"<$> Gastrointestinal tract (diarrheas)	10/23	43·5
<$>\raster="rg1"<$> Respiratory system (upper tract)	11/23	47·8
Failure to thrive	7/31	22·6
Fever of Unknown Origin (FUO)	7/31	22·6
Weakness and poor energy	3/31	9·7
Splenomegaly	2/31	6·4

Some of the presenting symptoms were omitted table 1 (when they were not the major complaint). However they were counted in the calculations of table 2.

### Laboratory Results

**Figure 2:**
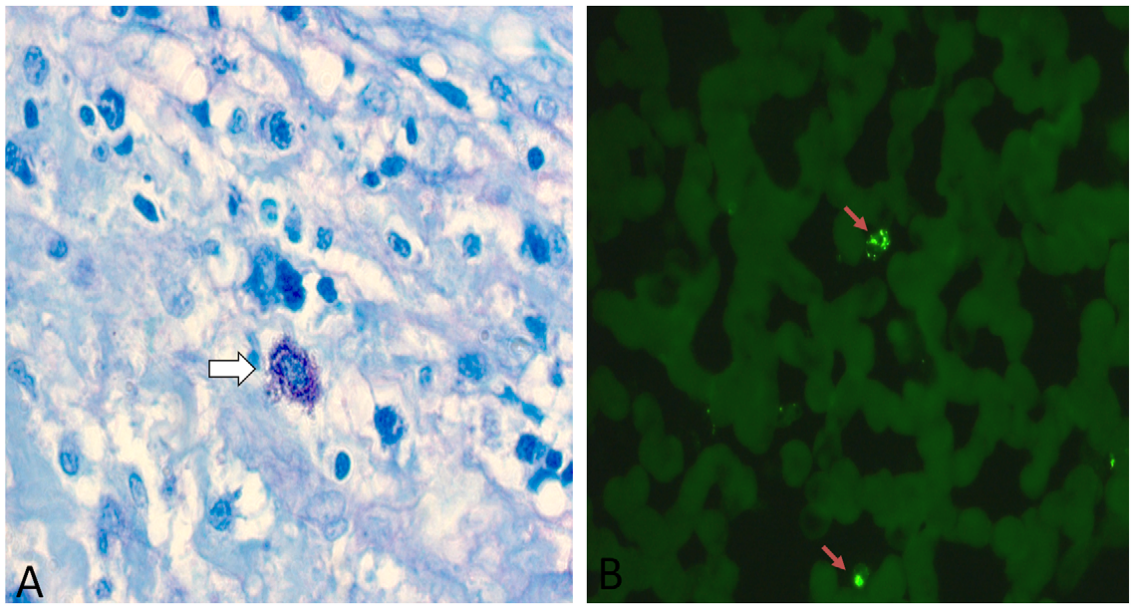
*Leishmania* parasites shown in lymphoid organs. (A) Giemsa stained section 40× mag. of a lymph node from patient 11 showing intracellular leishman bodies (purple in color) in the center of the photograph (white arrow). (B) Indirect immunohistochemistry using Fluorescein-labeled secondary antibody (FITC) and anti-leishmania primary antibody on a splenic aspirate from patient 9. *Leishmania* parasites are evident as bright green entities (red arrows, 40× mag.).

Reviewing the results of the laboratory studies on these 31 patients revealed that the CBC parameters were all borderline normal except in one case. The immunologic investigation by and large was normal (notably, negative *Leishmania* serology). IgG titers were upper normal in a few cases; only in one case, IgE levels were remarkably elevated with an eosinophil count ranging between 6 and 13% (on repeated testing). Worth mentioning that none of the 31 patients had positive skin test for *Leishmania* and tuberculosis. As for the organ biopsy, it did demonstrate the parasite but its efficacy depended on the sampled tissue. Figure 2 depicts two photomicrographs illustrating intracellular parasites on a section of lymph node (A), and on a splenic aspirate (B).

**Figure 3:**
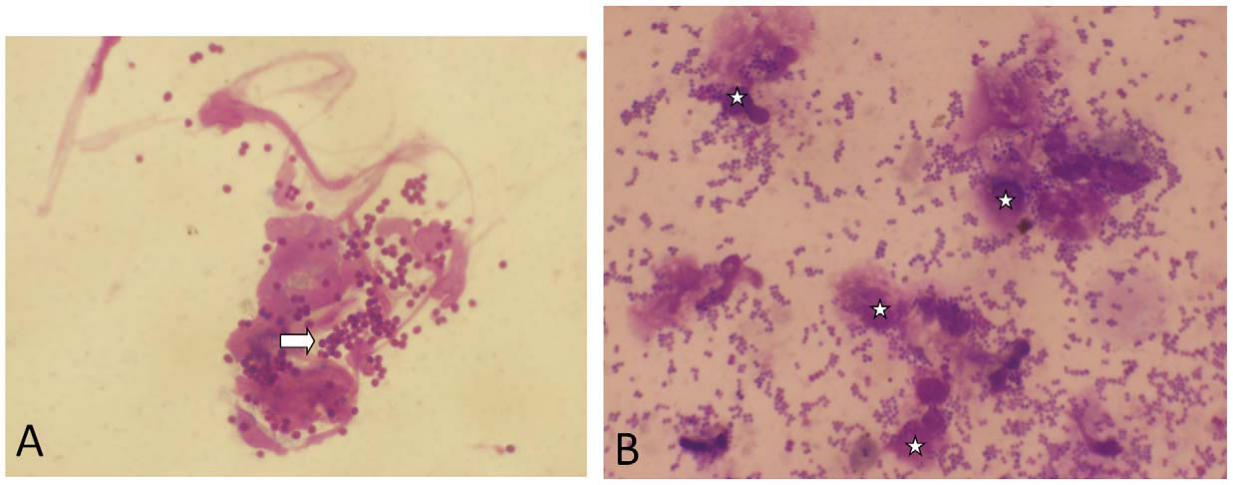
*Leishmania* parasites shown on Skin Windows. (A) A normal Skin Window showing intracellular (white star) and extracellular clumps (doublets) of *Leishmania* parasites, oil immersion lens 100× mag. (B) A normal Skin Window showing four monocytes in motion with many amastigotes in their cytoplasm (white arrows), oil immersion lens 100× mag.

The modified Skin Window was very efficient in demonstrating through its chronic phase the presence of the parasite within and without the monocytes in all 31 patients (Figure 3).

### Microscopy/Culture Results

Every time we obtained a biopsy for microscopic examination, part of it went into culture on NNN medium. The efficiency of demonstrating the parasite using microscopy and/or culture is summarized in table 3.

**Table 3:** Efficiency of different tissues in demonstrating and/or yielding cultures of *Leishmania* parasites.

Tissue	# of cases	Rate %
Whole blood	16/31	51·6
Buffy Coat	22/31	71·0
Plasma	1/31	3·2
Bone Marrow aspirate	10/31	32·2
Splenic aspirate	2/2	100
Skin Window	31/31	100

The best test for detecting the parasite microscopically was the Skin Window. The next in efficiency (culture and/or microscopy) was Buffy Coat, which revealed the presence of parasites in 71% of the cases. Whole blood followed. Bone marrow aspirate's yield was positive only in one third of the cases which could be a reflection of its depressed activity. Although obtaining a splenic aspirate entails a serious risk (the reason why we limited it to 2 patients), we still think it is a very efficient tissue in yielding parasites.

## Discussion

Kala-Azar has been established to present with a well defined clinical picture, but for the last few decades suspicion of having systemic leishmaniasis with a symptomatology different from the classically described, has been gaining greater and greater support. Such cases with a variable degree of illness were detected on the basis of positive serology [7]–[16].

In our series, the patients presented with symptoms typical of immune deficiency in the presence of a normal immune profile. Still they were found to harbor *Leishmania* organisms. In contrast to the reported sub-clinical cases among whom some subjects were totally symptom free while others had a milder form of the classical disorder, our cases presented with depressed immunity exhibiting symptoms pertaining to one or two target systems or organs.

A novel clinical picture of Visceral Leishmaniasis started to unfold. It consists of depressed immunity predisposing the subjects to infections with a wide spectrum of organisms (bacterial, viral and fungal). They presented with symptoms varying in type and severity. Although these secondary infections tend to resolve (with or without treatment), yet they invariably recurr. Thus in most patients and before referral, systemic anti-bacterial treatment was extensively used, sometimes in association with surgical interventions especially for skin abscesses and other tumorous growths. Anti-fungal agents were mostly added in cases with gastrointestinal complaints. Improvement or partial relief of symptoms arising from the superimposed infections was reported in most of our cases, but no complete cure was ever attained. Furthermore, it is important to note that none of the patients evolved to display the full fledged picture of Kala-Azar.

This form of atypical presentation is distinct from what has been described so far as sub-clinical illness meaning that it is not a lighter classical disease. This entity is novel in the sense that all patients were suspected to be immune-compromised and hence were sent to us for further immune function studies. Incidentally while performing the Skin Window to test the acute and chronic immune cellular responses to injury, we detected leishman bodies within and without the monocytes and some of the lymphocytes constituting the chronic phase of this reaction.

Whereas other investigators relied on immunological tests (purely serological) for proving the presence of the parasite in the candidate patients, we favored demonstrating it microscopically within any of the appropriate tissues involved. Although the spleen when enlarged seemed to be the ideal organ to reveal the parasite, yet the difficulty and the high risk implicated in its aspiration discourages most investigators from sampling it. Buffy Coat examination has obviously several advantages and was efficient in a good number of patients; however its efficacy drops the more the patient is anemic.

Besides, this set of data support the fact that systemic leishmaniasis in Lebanon and probably the area exists to a much greater extent than what we reported in our previous surveillance study [20]. Furthermore, the cases were detected in referrals from only two medical centers. Admitting the fact that the number of patients we are reporting is small for solid statistical analysis, still we think it may not be futile to detect a trend that could characterize such patients. So far the skin seems to be the organ most frequently targeted by these parasites. This is not surprising since these preferentially reside in cells of the hair follicles. Other systems may represent the shock organs being frequently affected by infections as happens in defective body defenses. As expected, it is the younger subjects (infants and children) who are the population at high risk.

Our major recommendation is to entertain the diagnosis of atypical leishmaniasis irrespective of age or country of patients presenting with depressed immunity, especially so whenever thorough investigation covering a large spectrum of diseases fails and multiple shotgun style therapies are tried without any success.
